# *In vivo* assessment of the effect of gel containing lactic acid and glycogen on vaginal microbiota and pH of asymptomatic women of reproductive age

**DOI:** 10.1371/journal.pone.0321737

**Published:** 2025-04-24

**Authors:** Adriana Bittencourt Campaner, Ana Carolina Alves Rosário Sica, Flavia Salomão d’ Avila Curi, Giulia Marchetti, Grazielle Suhett Dias, Bianca Luise Teixeira

**Affiliations:** 1 Department of Gynecology and Obstetrics, Santa Casa de São Paulo School of Medical Sciences, São Paulo, Brazil,; 2 BiomeHub Research and Development, Florianópolis, Santa Catarina, Brazil; Universidade dos Açores Departamento de Biologia: Universidade dos Acores Departamento de Biologia, PORTUGAL

## Abstract

Background

Vaginal dysbiosis is considered a risk factor for the development of gynecological and obstetric complications. This study aimed to evaluate the effects of vaginal gel containing lactic acid on vaginal pH, microbiota composition, and *Candida* species in asymptomatic women of reproductive age. Methods: 55 menstruating women, with no gynecological complaints, participated in the study, using a gel containing lactic acid twice a week, for 8 weeks. Assessments were conducted before and after the intervention, including measurements of vaginal pH, Nugent score, vaginal microbiota characteristics (alpha diversity index and Community State Types [CSTs] classification), and identification of *Candida* species. Results: At baseline, 36.4% of women exhibited CST type IV vaginal microbiota, followed by CST type III (30.9%), with vaginal pH values ranging from 4 to 5. After the intervention, there was a significant reduction in vaginal pH (p = 0.0057) and Nugent scores (p = 0.0047). Furthermore, a statistically significant decline was observed in the prevalence of unfavorable taxonomic groups, including *Prevotella amnii* and Bacterial Vaginosis Associated Bacterium 1, while vaginal lactobacilli remained unaffected. Despite these positive changes, the intervention did not result in a statistically significant alteration in microbial diversity or CST composition (p = 0.12). The gel was well-tolerated, with a low incidence of mild adverse effects reported. Conclusions: The lactic acid-containing gel demonstrated a significant reduction in vaginal pH and Nugent scores in asymptomatic women, indicating possible improvement in vaginal health, that needs to be confirmed in a placebo-controlled study. Although the intervention did not influence microbial diversity and the type of vaginal microbiota at the end of the study, it was able to reduce the prevalence of some taxonomic groups after intervention with the vaginal gel.

## Introduction

The human microbiome is a complex community of microorganisms that inhabit various regions of the body, including the intestines, skin, and vagina, along with their genomes and the surrounding environment. Studies have acknowledged that healthy individuals can exhibit differences in their microbiota composition, influenced by factors such as diet, living environment, host genetic characteristics and early microbial exposure, although the precise reasons for this diversity remain unclear [1].

The vaginal microbiota, a dynamic and complex ecosystem, plays a critical role in women’s health. Comprising over 250 bacterial species, it is predominantly populated by lactobacilli [2]. These microorganisms protect the vaginal environment through mechanisms such competing with other microorganisms for nutrients and adhesion sites, reducing vaginal pH via the production of organic acids (mainly lactic acid), modulating the local immunity and producing antimicrobial substances, such as bacteriocins. Together, these actions create a robust defense against colonization by opportunistic pathogens, ensuring a balanced and healthy microenvironment [2,3].

According to a pioneering study by Ravel et al [4], the vaginal microbial community in asymptomatic reproductive-age women can be classified into five Community State Types (CSTs I–V). CSTs I, II, III, and V are dominated by *Lactobacillus crispatus*, *L. gasseri*, *L. iners*, and *L. jensenii*, respectively, whereas CST IV exhibits high microbial diversity, characterized by aerobic and obligate anaerobic bacteria such as *Prevotella*, *Dialister*, *Atopobium*, *Gardnerella*, *Megasphaera*, *Peptoniphilus*, *Sneathia*, *Eggerthella*, *Aerococcus*, *Finegoldia*, and *Mobiluncus* [4,5]. CST IV, while observed in healthy women, is associated with higher Nugent scores, representing intermediate bacterial vaginosis (BV) and bacterial vaginosis (Nugent scores 4–6 and 7–9, respectively) [6,7].

Various factors influence the vaginal microbiota throughout a woman’s life, including age, ethnicity, sexual activity, hormonal fluctuations, pregnancy, menstruation and menopause, genital infections and personal hygiene [2,8,9]. Dysbiosis, or the imbalance of vaginal microbiota, is recognized as a risk factor for gynecological and obstetric complications, contributing to conditions such as pelvic inflammatory disease, spontaneous abortion, preterm birth, endometritis, infertility, increased susceptibility to sexually transmitted infections (STIs), and cervical carcinogenesis. [10-13]

Evidence suggests that lactic acid, at physiological concentrations, can lower vaginal pH, thereby reducing the growth of anaerobic bacteria and fostering the colonization of lactobacilli in the vaginal environment [14-16]. Lactic acid exists in two isomeric forms, D (-) and L (+), both of which are present in vaginal fluid. The proportions of these isomers depend on the predominant lactobacillus species. Notably, D (-)-lactic acid-producing lactobacilli are believed to have a protective role in the vaginal environment. The specific balance of these lactic acid isomers may affect the host’s susceptibility to infections, underscoring the importance of host-microbiota interactions. [14–16].

In this context, the present study aimed to evaluate the effects of a lactic acid-containing vaginal gel on the pH, vaginal microbiota composition, and Candida species in healthy women.

## Materials and Methods

This pilot, open, non-randomized, before-and-after study was conducted from August 2022 to July 2023 at the Irmandade da Santa Casa de Misericórdia de São Paulo within the Lower Genital Tract Pathology and Colposcopy Outpatient Clinic. A total of 55 participants, aged 19 to 50 years and in menacme period, were recruited through convenience sampling, based on inclusion and exclusion criteria. They were randomly selected at the outpatient clinic.

The sample size was calculated using microbial diversity as the primary outcome, with a mean Shannon index of 3.6 and a standard deviation of up to 1.2, based on previous data from *Human Microbiome Project*. The study aimed to detect an absolute variation of 0.45 in the Shannon index with 80% statistical power, using a two-sided, paired t-test with a significance level of 5%. We considered that zero patients would drop out from the study.

Inclusion criteria required participants to be menstruating women aged 18-50 years with no gynecological complaints, a normal gynecological examination and no evidence of vaginal discharge or local lesion. Participants were also required to understand the study’s objectives risks, and adverse effects, and agree to comply with the full protocol. None of the participants had a history of vaginal dysbiosis, sexually transmitted infections or related symptoms.

Exclusion criteria included known hypersensitivity to the formula components; pregnancy or breastfeeding; menopause (defined as women aged ≥ 50 years with amenorrhea for at least one year); lack of prior sexual activity; clinical signs of genital infections; prior treatment for cervical intraepithelial neoplasia or cervical carcinoma; recent gynecological procedures (within the last month) or history of using intravaginal douching; intravaginal or systemic antimicrobial or antifungal treatment within 4 weeks prior to study inclusion; immunosuppression (due to drugs or diseases); current or recent diagnosis of neoplasms; decompensated endocrinopathies; participation in experimental studies or ingestion of experimental drug within the last 12 months. There were no restrictions regarding contraception, except for intrauterine device users.

Participants were requested to abstain from using antibiotics and antifungals (vaginal or systemic), immunosuppressive medications (e.g., corticosteroids, cyclosporine), probiotics, or any vaginal products during the study. They were also required to avoid pregnancy and vaginal douching during the study period. Non-compliance with these instructions would result in exclusion from the study. Participants were allowed to maintain regular sexual activity, with the choice to use or not use condoms, and were advised to abstain from using the gel during their menstrual period.

### Ethics statement

The study was approved by the Institutional Review Board of Irmandade da Santa Casa de Misericórdia de São Paulo under the following registration number: CAAE: 60490722.4.0000.5479 (uploaded). A written consent form was signed by all women that agreed to participate in this study.

### Description of clinical procedures and acid gel application

Participants underwent a series of procedures, beginning with a detailed medical history followed by a comprehensive physical and gynecological examination. Measurement of vaginal pH was taken, and vaginal specimen was collected for both Nugent scoring and microbiota sequencing (to assess alpha diversity index and CST classification). After these clinical procedures, each participant received a box containing the study medication and was instructed in its use. Specifically, participants were advised to insert the applicator filled with 5g of gel into the vagina at bedtime. The gel formulation contained lactic acid (225 mg per application), glycogen (5 mg per application), hypromellose, propylene glycol, sodium benzoate, sodium hydroxide and purified water. This product, commercially available in Brazil as Relactagel ® (Marjan Farma, São Paulo, Brazil) has been approved by the National Health Surveillance Agency (Anvisa), under for indications such as genital hydration and maintenance of vaginal pH. Glycogen was associated with the formulation to support the maintenance of lactobacilli. Participants were instructed to apply the gel twice a week, for a period of 8 weeks. To ensure adherence, they were provided with a card to track their application schedule. A follow-up appointment for clinical evaluation and post-treatment specimen collection was scheduled for 8 weeks (± 2 weeks) after the first application of the vaginal gel. At this visit, several procedures were performed, including a clinical assessment, review of the participant’s application card and investigation of adverse events. A complete physical and gynecological examination, measurement of vaginal pH, and vaginal specimen for Nugent scoring and microbiota sequencing (alpha diversity index and CST classification).

Participants’ acceptability of the lactic acid-containing gel was assessed using a questionnaire comprising 6 questions: five multiple-choice questions and one open-ended question. Topics included lubrication, moisture, dryness of the intimate area, comfort during sexual intercourse and the likelihood of future use of the product (detailed in the supplementary section). Regarding adverse events, participants were encouraged to freely report the signs and symptoms they experienced while using the gel.

### Vaginal pH measurement and vaginal specimen collection

The participant was positioned in a gynecological position, and a sterile speculum was inserted into the vagina. Sample collections were performed regardless of the menstrual cycle phase, excluding the menstrual period. The procedures were conducted in the following chronological order:

- Measurement of vaginal pH: the pH strip (MQuant® -Supelco) was placed on the right vaginal wall, at the middle third, in direct contact with the vaginal mucosa for approximately one minute. The pH was then recorded using the strip’s scale (range 0-14; measured in intervals of 1).
- Specimen collection for Nugent scoring: vaginal material was collected from the vaginal cul-de-sac using a sterile cotton swab. The sample was rolled on a glass slide (without fixation) and sent to the institution’s Pathology Department for staining and analysis. The Nugent score was calculated by assessing for the presence of (i) large Gram-positive rods (*Lactobacillus* morphotypes; decrease in Lactobacillus scored as 0 to 4), (ii) small Gram-variable rods (*Gardnerella vaginalis* morphotypes; scored as 0 to 4), and (iii) curved Gram-variable rods (*Mobiluncus spp*. morphotypes; scored as 0 to 2). The results were interpreted as: 0 to 3: normal; 4 to 6: intermediate; 7 to l0: bacterial vaginosis.
- Specimen collection for microbiota sequencing: vaginal material was collected from the vaginal cul-de-sac using a sterile swab (Hydraflock®, Puritan, USA), which was then placed into a transport bottle containing a proprietary stabilizing solution from BiomeHub (Brazil). This solution halted biological and metabolic processes, preserving only the DNA of the organisms present in the sample. Samples were kept at room temperature and sent to BiomeHub for analysis. The interval between sample collection and arrival at the laboratory was less than 30 days.

Participants were advised to abstain from sexual intercourse for 72 hours prior to sample collection, and collections were performed on non-menstrual days to ensure sample integrity.

### Molecular analysis to assess the vaginal microbiota

For vaginal microbiome samples, DNA extraction was conducted using a modified protocol of the QIAamp DNA Blood Mini (QIAGEN, USA). Bacterial identification from vaginal samples was achieved through high-throughput sequencing of the V3/V4 regions of the 16S rRNA gene. Fungal identification was performed, using universal primers targeting the ITS1 region of the internal transcribed spacer.

The library preparation involved two PCR amplification steps. The first PCR used primers for the V3/V4 region of the 16S rRNA gene (341F CCTACGGGRSGCAGCAG and 806R GGACTACHVGGGTWTCTAAT) and for the ITS gene (ITS1 GAACCWGCGGARGGATCA and ITS2 GCTGCGTTCTTCATCGATGC). Universal primers for the 16S gene and for the ITS1 region were employed for bacterial and fungal identification, respectively. These primers were designed with adapters based on the TruSeq approach (Illumina Inc., USA).

The second PCR inserted index sequences into the libraries, enabling sample identification. The final PCR reaction was purified using a magnetic bead-based protocol (AMPureXP, Beckman Coulter, USA). The purified libraries were then pooled into a single library pool for quantification. Quantification was performed by qPCR using the Collibri Library Quantification Kit (Invitrogen, United States).

Sequencing was conducted on a MiSeq Sequencing System equipment (Illumina Inc., USA) using the V2 kit with 300 cycles and single-end sequencing, according to the manufacturer’s specifications. The sequencing runs achieved a target coverage of 30,000 reads per sample for all samples analyzed.

### Sequencing data assessment

The obtained sequences (reads) were processed using a bioinformatics pipeline developed by BiomeHub (Encodetools, BiomeHub, Brazil), based on the methodology described by Cruz et al. [17] Initially, the data were demultiplexed Illumina`s software (bcl2fastq v2.2.0), and subsequently evaluated by the BiomeHub pipeline for the presence of the forward primer at the beginning of each sequence. A maximum of one mismatch in the primer region was allowed; sequences failing to meet this criterion were discarded. Following these step primers were trimmed, and the accumulated sequence error was calculated by converting the Q score of each nucleotide into error probability, summing these probabilities, and dividing by the sequence length. Sequences with an accumulated error greater than 0.35% were excluded.

Sequences that passed the quality filter were further processed with Deblur v1.1.0 software to remove potential sequencing errors. Those with 100% identity and coverage were grouped into Amplicon Sequence Variants (ASVs), internally referred as oligotypes. The presence of chimeras was then assessed using VSEARCH v2.13.6 software. An additional frequency filter was applied to exclude ASVs with a frequency below 0.2% within the sample. A negative control filter was also employed for low biomass samples; any ASV detected in negative controls was assessed against the analyzed samples and removed from the results if its frequency was less than twice the values observed in the controls. The remaining ASVs from these qualification processes would then proceed to the taxonomic classification and analysis stages.

Taxonomic classification was performed using BLAST search tool against BiomeHub reference databases (encoderef16s_rev8_210105 and encoderefHerBiome_rev5_221115, BiomeHub, Santa Catarina, Brazil). These databases, constructed and manually curated, include complete and partial genome sequences (draft) of bacteria (including clinically relevant species), and fungi, all of them obtained from NCBI. Taxonomies were assigned to each ASV using an LCA (Lowest Common Ancestor) algorithm, which assigns the ASV to the lowest common taxonomic rank when multiple references share the same coverage and identity (e.g. genus, family, order). The vaginal microbiome was subsequently classified into five CSTs as described in previous study [4].

Alpha diversity was calculated using the estimate richness function in the *phyloseq* R package incorporating metrics such as Shannon, Simpson, and richness. The datasets generated in this study are publicly available in an online repository. The names of the repository and accession number can be found at: https://www.ncbi.nlm.nih.gov, BioProject PRJNA1102271.

### Statistical analysis

All collected data was entered into an Excel spreadsheet for initial descriptive analysis. Statistical analyzes were conducted using R software, version 4.3.1 (R Core Team, 2020). To evaluate alpha diversity indices (Shannon, Inverse Simpson, and richness), vaginal pH, and Nugent scores, Wilcoxon signed-rank tests were applied at a 5% significance level. These rank-based tests were employed to assess general distributional differences (i.e., instead of simple mean differences). No normality tests were performed.

Differential abundance analysis was conducted using the R package MicrobiomeStat employing the LinDA method, with a random intercept per participant [18]. Paired analyses of categorical outcomes, such as the presence of *Candida* and vaginal microbiota types, were performed using Stuart-Maxwell and McNemar tests, as appropriate.

To ensure robust statistical control, global error rate adjustments were implemented: The Bonferroni procedure was applied to comparisons of pH and Nugent scores to manage the Family-Wise Error Rate (FWER). For microbiota data, the Benjamini-Hochberg procedure was used to control the False Discovery Rate (FDR) at 10%.

## Results

The study included 55 women in the reproductive period aged 19- 50 years. The majority were non-smokers (83.6%) and sexually active during the period of medication use (85.4%), with 63.4% not using condoms. All participants reported having only one sexual partner during the study. At the time of the initial specimen collection (T=0), 36.36% of the women exhibited CST type IV vaginal microbiota, followed by CST type III (30.9%) and CST type I (25.4%). The remaining participants had vaginal microbiota CST types II (3.6%), CSTV (1.8%) or unclassified microbiota (1.8%). Regarding vaginal pH, the majority had values of 4 (43.6%) or 5 (47.3%). For Nugent scores, 49.09% had a score of 0, indicating a *Lactobacillus*-dominated microbiota, while 34.5% had a score of 8, consistent with bacterial vaginosis (Table 1).

**Table 1:** Characteristics of the study cohort.

Parameters	T=0	T=1	P value
**Microbiota type**			
I	14 (25.4%)	20 (36.4%)	
II	2 (3.4%)	0 (0.0%)	
III	17 (30.1)	16 (29.1%)	p = 0.12 (not significant).
IV	20 (36.4%)	17 (30.9)	
V	1 (1.8%)	2 (3.6%)	
Unclassified microbiota	1 (1.8%)	0 (0.0%)	
**pH**			
4	24 (43.6%)	35 (63.6%)	p = 0.0057 (significant)
5	26 (47.3%)	19 (34.5%)	
6	5 (9.1%)	1 (1.8%)	
**Nugent scores**			
Non Nugent-BV (0-3)	31 (56.4%)	42 (76.4%)	p = 0.0047(significant)
Intermediate (4-6)	5 (9.1%)	3 (5.5%)	
Nugent-BV (7-9)	19 (34.5%)	10 (18.2%)	

Initial specimen collection (T=0); second specimen collection (T=1); Nugent score: l*actobacillus* dominant (Nugent 0 – 3), Intermediate (4–6) and BV (7–9).

After 8 weeks of intervention, in the second specimen collection (T=1), 36.4% of women had their vaginal microbiota classified as type I, followed by type IV (30.9%), type III (29.1%) and type V (3.6%). Concerning pH, after treatment, the majority of women presented a value of 4 (63.6%), followed by a value of 5 (34.5%). As for Nugent scores, the majority of women had a score of 0 (72.7%), followed by a score of 8 (18.2%) (Table 1).

### Vaginal microbiota profile before and after treatment

**Fig. 1.**
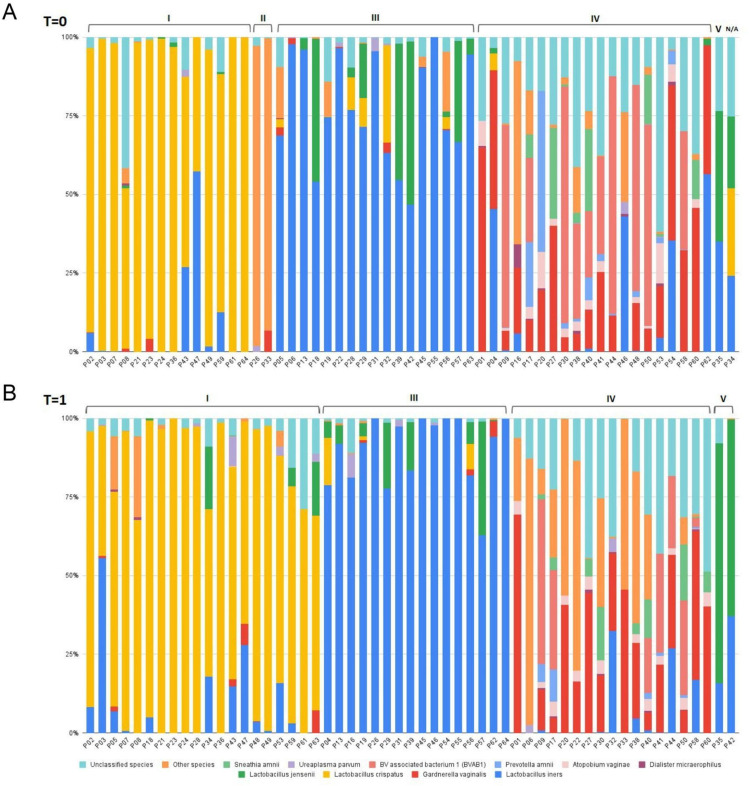
Graphical representation of proportion of bacteria in each sample, per participant, before (T **=0) (A) and after (T=1) (B) the use of the vaginal gel.** The 10 most prevalent species were highlighted and other less frequent species were grouped.

The profile of bacterial species identified in each participant’s vaginal microbiota by 16S amplicon sequencing, both before and after using the vaginal gel, is presented in Fig. 1. A total of 59 bacterial species were identified; the five most prevalent species were *L.iners* (presented in 68.2% of samples), *Gardnerella spp.*(48.2% of samples), *L.crispatus* (41.8% of samples), *L.jensenii* (28.2% of samples) and *Fannyhessea vaginae*, previously known as *Atopobium vaginae* (26.4% of samples).

### Assessment of bacterial diversity

**Fig. 2.**
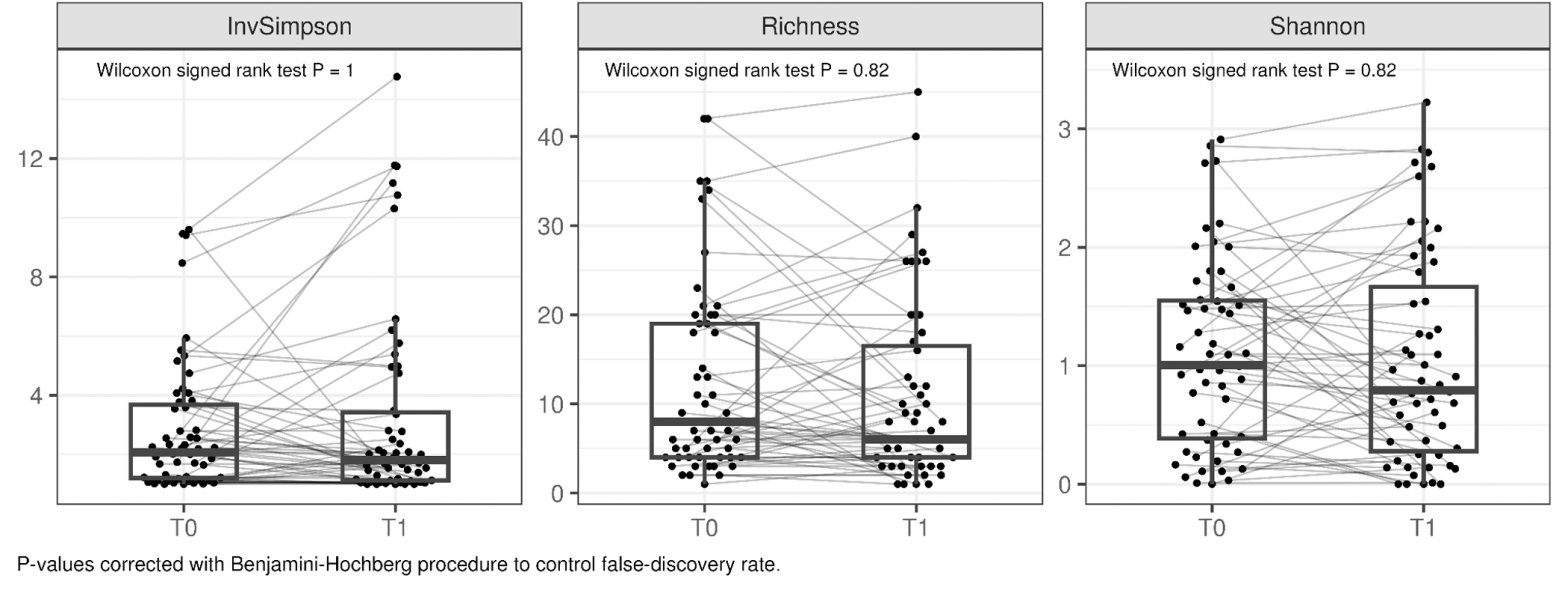
Comparison of participants’ microbiota before and after the intervention in terms of alpha-diversity, with 3 different metrics (Inverse Simpson, richness and Shannon indices). In all cases, no statistically significant differences were observed before and after treatment.

We assessed Inverse Simpson, richness and Shannon indices as main alpha-diversity metrics per sample and the results are presented in Fig. 2. However, no significant changes were observed between alpha-diversity profiles and the use of gel containing lactic acid.”

### Bacterial differential abundance assessment

**Fig. 3.**
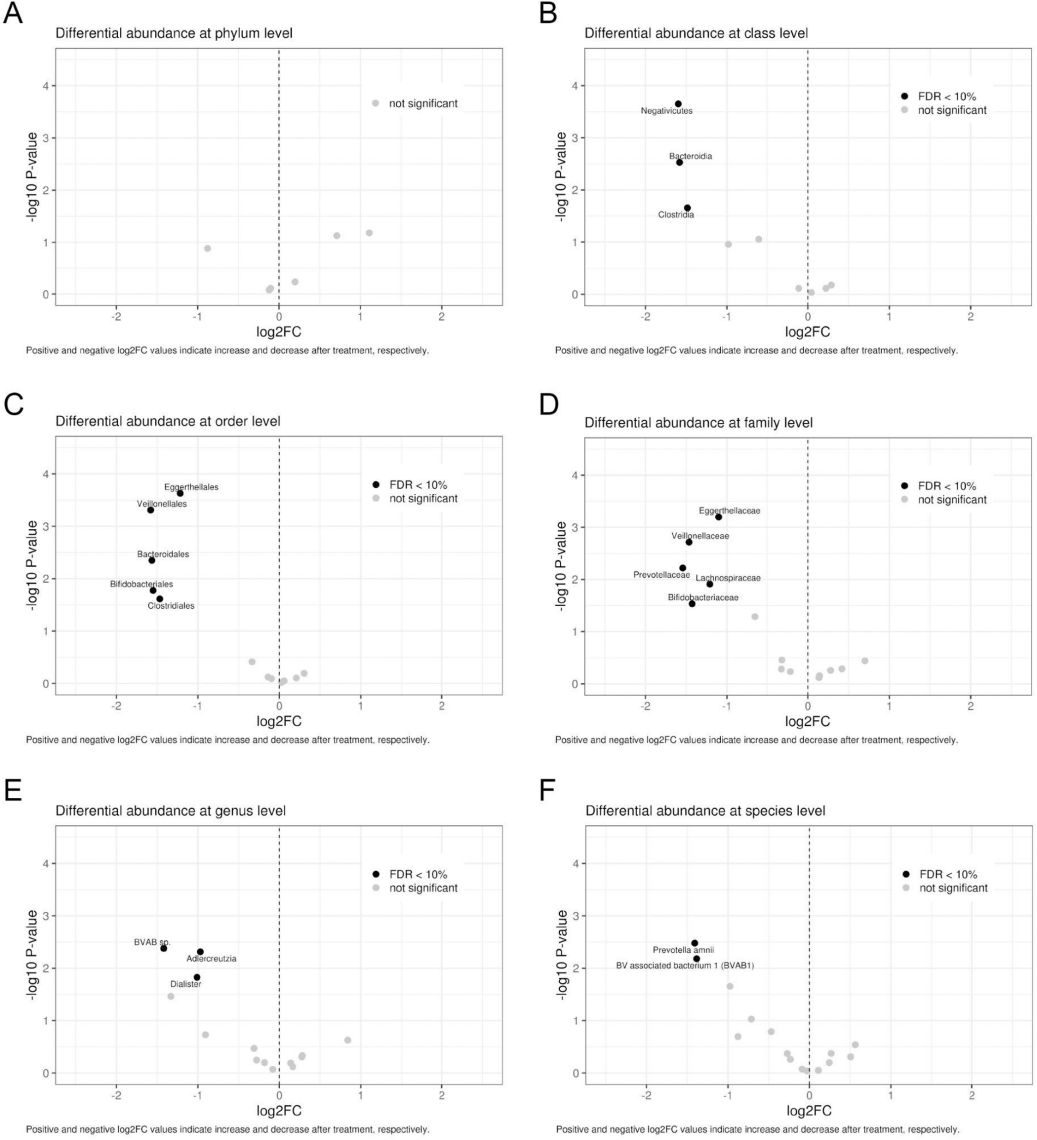
Results of differential abundance assessment expressed as Log2 Fold Change (Log2FC) for comparison between different taxonomic groups after the intervention. The Volcano plots illustrate the results of differential abundance assessment at the levels of phylum (A), class (B), order (C), family (D), genus (E), and species (F). Black dots indicate FDR (False discovery rate) lower than 10%. Positive and negative Log2FC values indicate an increase or decrease after treatment, respectively.

The association between bacterial abundance and intervention was assessed using the LinDA method, with results presented in Fig. 3. Some taxonomic groups were differentially abundant after the intervention, as is the case of the classes *Negativicutes*, *Bacteroidia* and *Clostridia*; of the orders *Eggerthellales*, *Veillonellale*s, *Bacteroidales*, *Bfidobcteriales* and *Clostridiales*; from the families *Eggerthellaceae*, *Veillonellaceae*, *Prevotellaceae*, *Lachnospiraceae* and *Bifidobacteriacea*; from the genera *BVAV sp*., *Adlercreutzia* and *Dialister*; and the species *Prevotellaamnii* and *BV associated bacterium 1* (BVAB1). All these taxonomic groups depicted a statistically significant reduction in their prevalence after intervention with the vaginal gel which are considered unfavorable ones for the vaginal health. Importantly, the medication had no negative effect on vaginal lactobacilli.

In the differential abundance charts, only the taxonomies showing statistically significant differences between time points are represented. Since lactobacilli were not differentially abundant between the time points, they are not highlighted in Fig 3, just like other taxonomies that showed no difference in abundance.

### Assessment of vaginal microbiota type before and after the intervention

Regarding the classification of microbiota types before and after the intervention, 23.6% of women were classified as type I in the beginning of the study and remained type I after the intervention. Similarly, 23.6% were classified as type IV and maintained this classification after, while 16.4% were initially classified as type III and remained as type III after using the gel. A small number of participants experienced changes: three women (5.4%), initially classified as type III, and one woman (1.8%) classified as type II were reclassified as type IV following the intervention.

**Fig. 4.**
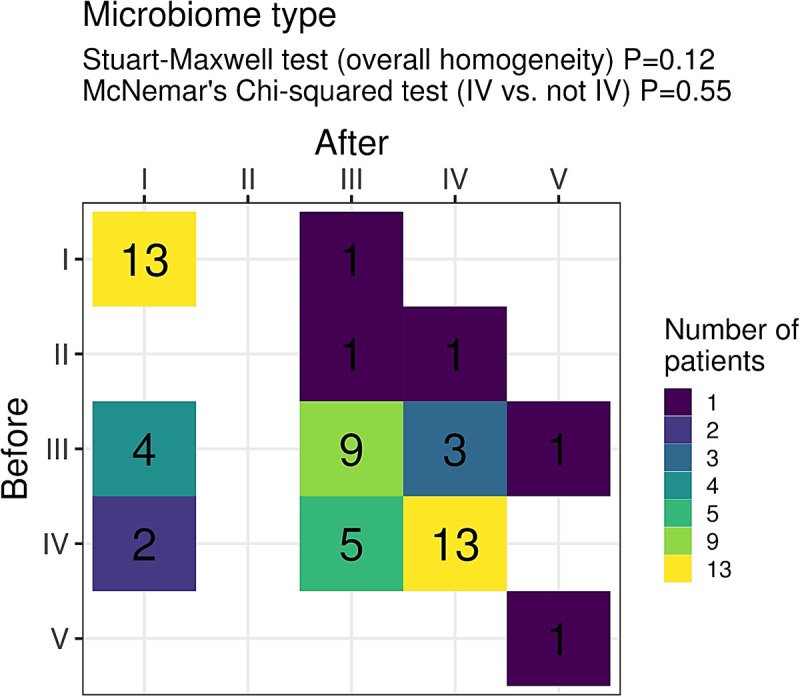
Assessment of the type of microbiota before (T **=0) and after (T=1) the use of the vaginal gel.** There was no statistically significant difference between the type of microbiota before and after the intervention (p value =0.12).

Despite these observation, there was no statistically significant difference in microbiota classification before and after the intervention (p=0.12), nor between type IV microbiota and the other types (p=0.55) (Fig. 4). This indicates that the use of lactic acid-containing gel did not directly alter the vaginal CST in the analyzed population. Additionally, there was no age-related difference in the distribution of vaginal microbiota types or in the effectiveness of the vaginal gel.

### Assessment of pH before and after the intervention

Regarding pH values before and after the intervention, 38.2% of the women initially had a pH of 4, which remained unchanged, and 21.8% initially had a pH of 5 and also retained the same value after using the gel. However, 23.6% of women with an initial pH value of 5 and 1.8% of those who had a pH of 6 experienced a decrease in pH to 4 following the intervention.

Overall, a statistically significant reduction in the pH value was observed after the intervention (p =0.0057) (Table 2). This finding suggests that the application of the lactic acid-containing gel might reduce pH values in the analyzed population.

**Table 2.** pH assessment before (T=0) and after (T=1) use of the vaginal gel. A statistically significant reduction in the pH value was observed after the intervention (p value =0.0057).

pH (T=0→T=1)	N=55
**pH4 → pH4**	21 (38.2%)
**pH4 → pH5**	3 (5.4%)
**pH5 → pH4**	13 (23.6%)
**pH5 → pH5**	12 (21.8%)
**pH5 → pH6**	1 (1.8%)
**pH6 → pH4**	1 (1.8%)
**pH6 → pH5**	4 (7.3%)

### Nugent score before and after the intervention

Regarding the Nugent score before and after the intervention, 47.3% of women initially had a score of 0, which remained unchanged, and 16.4% had a score of 8 and also retained the same value after using the gel (see supporting information). Despite these consistent individual scores, a statistically significant decrease in Nugent scores was observed across the population after the intervention (p =0.0047) (Table 3). This finding indicates that the application of the lactic acid-containing gel effectively reduced the Nugent score values in the assessed population.

**Table 3:** Analysis of the Nugent score before (T=0) and after (T=1) the use of the vaginal gel. Number of participants and respective percentages of Nugent scores before and after the intervention. A statistically significant decrease in Nugent scores was observed after the intervention (p value =0.0047).

Nugent (T=0→T=1)	N=55
**0 → 0**	26 (47.3%)
**0 → 8**	1 (1.8%)
**1 → 1**	2 (3.6%)
**1 → 0**	2 (3.6%)
**5 → 5**	1 (1.8%)
**5 → 0**	4 (7.3%)
**8 → 8**	9 (16.4%)
**8 → 0**	8 (14.5%)
**8 → 5**	2 (3.6%)

The study included women from white, brown, and black racial groups. Due to the small number of participants in each group, it was not possible to identify statistically significant differences in pH, Nugent scores, or CSTs among these groups.

### Assessment of *Candida albicans* presence before and after the intervention

Although the study was conducted with women without vaginal symptoms, *Candida albicans* was detected in 9 women (16.36%) vaginal microbiota prior to the intervention (T0). Despite investigating all *Candida* species, only *C. albicans* was identified. Among the 9 women initially positive for *C. albicans*, 6 (66.7%) no longer exhibited the fungus in their vaginal microbiota after using the vaginal gel. Conversely, 2 women (3.6%) who were negative for *Candida albicans* before the intervention (T0) exhibited the presence of this fungus after the intervention. However, no statistically significant difference was detected in the prevalence of *Candida albicans* before and after the intervention (p value =0.29).

### Acceptability of gel containing lactic acid

The participants’ acceptability of the lactic acid-containing gel was evaluated through a questionnaire addressing lubrication, moisture, dryness of the intimate area, comfort during sexual intercourse and the likelihood of future use of the product. It was observed that the majority of women declared an improvement in lubrication/moisture of the intimate area during sexual intercourse (70.9%, 39 out of 55 women), while the majority declared there was no change in the dryness of the intimate area (69.1%, 38 out of 55 women) and in comfort during sexual intercourse (41.8%, 23 out of 55 women) after using the vaginal gel. However, the majority of women expressed a willingness to use the product in the future. (81.8%, 45 out of 55 women).

### Assessment of adverse events

The adverse events informed by the participants were burning and itching, which were considered mild and did not result in abandonment and/or discontinuation of the use of the product. Of the 55 participants, 30 women (54.5%) reported feeling at least one episode of burning after applying the gel. However, the incidence of burning was reported in only 22.7% of total applications (n=880). Regarding itching, 11 women (20%) reported feeling at least one episode of itching after applying the gel. The incidence of itching, however, was reported in only 5.5% of total applications (n=880). When assessing these results, no association was identified between the presence of reported adverse events and the type of vaginal microbiota of the participants prior to using the vaginal gel.

## Discussions

In this study we evaluated the effects of a vaginal gel containing lactic acid on the vaginal pH, vaginal microbiota composition and the presence of Candida species in healthy women. After the intervention, a significant reduction in vaginal pH values and Nugent scores was observed. However, there was no statistically significant changes in the type of microbiota. Additionally, a statistically significant reduction was observed in the prevalence of some unfavorable taxonomic groups, including the species *Prevotella amnii* and *Bacterial vaginosis associated bacterium 1*. No negative impact on vaginal lactobacilli was observed.

The vaginal microbiota (VMB) accounts for approximately 9% of the total human microbiota, forming a mutualistic relationship with the host (vaginal environment), protecting it from potentially pathogenic agents. [4.8] Lactobacilli are known to provide protection against pathogens in several ways, including the production of lactic acid (LA) and bacteriocins, where LA is believed to play a central role in vaginal environment defense. [19]

Surprisingly, several anaerobic bacteria have been documented as predominant in asymptomatic women, indicating that *Lactobacillus* dominance could not necessarily be a feature of healthy vaginal microbiota. [2,4,8] This fact can be verified in our study: when evaluating the microbiotas of 55 asymptomatic women, we observed that the most frequent type of VMB observed was CST type IV (36.36%), followed by CST type III (30.91%), and CST type I (25.45%). However, this finding was unexpected, as the studied population had no clinical abnormalities, and *Lactobacillu*s dominance was anticipated. Nevertheless, these women could be considered asymptomatic carriers of bacterial vaginosis. The only way to determine whether a non-Lactobacillus-dominated microbiota can be considered healthy would be to assess the absence of adverse sequelae and/or elevated local inflammation—factors beyond the scope of this study.

This observation reinforces the complexity of vaginal dysbiosis, which is often characterized as VMB not dominated by lactobacilli. However, this condition can be either physiological or pathological, depending on the interaction of metabolic and microbial factors [8,9]. Studies have linked vaginal dysbiosis to an increased susceptibility to sexually transmitted infections, including HIV, as well as other complications such as vaginal discharge, pelvic inflammatory disease [11,20], complications in pregnancy [21], infertility [13], negative impact on assisted reproduction techniques [22], and even an increased risk of cervical carcinogenesis [12].

VMB in a dysbiotic state is characterized by low lactic acid production, elevated vaginal pH (>4.5) and increased production of biogenic amines. These conditions promote the growth of pathogenic bacteria and inhibit *Lactobacillus* species. In this dysbiotic environment, there is infiltration of CD4+ T cells and the production of cytokines that are cytotoxic to epithelial cells. This not only supports the growth of pathogenic anaerobes but also diminishes the local immune response. [23]

In this context, several studies have established that lactic acid in physiological concentrations can lower vaginal pH, inhibit the growth of anaerobic bacteria and promote the colonization of the vaginal environment by lactobacilli, thereby helping to maintain a healthy vaginal microbial environment. [24-29] For instance, O’Hanlon et al [24] demonstrated the antimicrobial action of lactic acid on BV-associated bacteria and vaginal lactobacilli *in vitro*, under anaerobic growth conditions, similarly to the hypoxic environment of the vagina. Their findings showed that lactic acid at physiological concentrations inactivated all tested BV-associated bacteria and had no detectable effect on vaginal lactobacilli. It is important to state that these are “in vitro” or “ex vivo” studies and the findings of these studies are “predicted” to maintain an optimal vaginal microbial environment.

Our study corroborates these findings by demonstrating a significant reduction in vaginal pH values and Nugent scores in the population analyzed following the use of a vaginal gel containing lactic acid. Although the intervention did not influence microbial diversity and the type of vaginal microbiota at the end of the study, it effectively reduced the prevalence of certain unfavorable bacterial taxonomic groups after intervention. Specifically, reductions were observed in bacteria from the classes: *Negativicutes*, *Bacteroidia* and *Clostridia*; from the orders *Eggerthellales*, *Veillonellales*, *Bacteroidales*, *Bfidobcteriales* and *Clostridiales*; from the families *Eggerthellaceae*, *Veillonellaceae*, *Prevotellaceae*, *Lachnospiraceae* and *Bifidobacteriacea*; from the genera *BVAV sp*., *Adlercreutzia* and *Dialister*; and the species *P.amnii* and *BV associated bacterium 1* (BVAB1). These reductions indicate a potential improvement in vaginal health, as these taxa are considered detrimental to a healthy vaginal environment.

In the present study, no negative influence on local lactobacilli was observed. This demonstrates that the application of the lactic acid-containing gel positively influenced parameters related to maintaining a balanced and healthy vaginal environment in asymptomatic women. Furthermore, the gel had good acceptability, with a low incidence of mild adverse effects. Interestingly, while the Nugent score significantly decreased, this change was not accompanied by modifications in the community state type (CST). A longer duration of treatment might potentially lead to CST alterations. Hearps et al [25], in an *in vitro* study, determined that lactic acid elicited an anti-inflammatory response in human cervicovaginal epithelial cells and inhibited the production of pro-inflammatory mediators, especially those associated with HIV infection. Similar findings were also observed by Tyssen et al [26], Manhanzva et al [27], Delgado-Diaz et al [28]. Another study [29] observed that lactic acid from the vaginal microbiota increased the integrity of the cervicovaginal epithelial barrier, promoting the expression of tight junction proteins; these data suggest that lactic acid could help prevent pathogens invasion that may cause sexually transmitted infections. The studies described above (*in vitro*) assessed changes in local physiology and biochemistry caused by the application of lactic acid. However, despite the importance of these findings, this was not the scope of our study.

Plummer et al [30], in a systematic review, assessed the efficacy of intravaginal lactic acid-containing products on BV cure and their impact on vaginal microbiota composition. The authors concluded that there is a lack of high-quality evidence to support the use of lactic acid-containing products for BV cure or vaginal microbiota modulation. Although our study included only asymptomatic women, some of them presented dysbiotic microbiota. Although the intervention did not influence microbial diversity and the type of vaginal microbiota at the end of the study, it was able to reduce the prevalence of some taxonomic groups after intervention with the vaginal gel.

Some other trials used *Lactobacillus* as vaginal gel and studied its effects on vaginal pH, microbiome and urogenital symptoms. Oerlemans et al [31] compared a *Lactobacillus*-based vaginal gel to fluconazole for treating vulvovaginal candidiasis. Both treatments resulted in similar fungal concentrations, though fluconazole reduced endogenous *Lactobacillus* counts. These findings could be observed in our study, where 6 out of 9 women (66.7%) who were initially positive for C. albicans no longer exhibited the fungus in their vaginal microbiota after using the vaginal gel. Yoshikata et al [32] evaluated the effects of Lactobacillus-containing feminine hygiene products (group 1) and feminine hygiene products associated with vaginal gel (group 2), both on vaginal microbiome in pre and postmenopausal women. Both treatments reduced vaginal pH and pathogenic microbiota compared to the control group, with more significant effects observed in postmenopausal women using the gel.

Shen et al [33] investigated the effect of a postbiotic gel in 50 patients with bacterial vaginitis (BV). The results showed that applying the gel improved the symptoms of BV, by improving vaginal secretions and increasing the relative abundance of *Lactobacillus* compared to baseline. This effect was also observed in the present study, where a significant reduction in the Nugent score (p=0.0047) was noted after the intervention, suggesting an improvement in vaginal microbiota quality.

Over the counter lactic acid gels on the market are typically used daily for 7 days generally as a treatment for bacterial vaginosis. However, in this study we opted for a twice-weekly dosing schedule over 8 weeks to maintain vaginal pH in asymptomatic women, rather than to treat any pre-existing condition. This methodology was chosen based on prior discussions with the investigators and in accordance with their previous clinical experiences. Despite some participants included in the study presenting vaginal dysbiosis, the study demonstrated beneficial effect of the medication, with a reduction in pH and Nugent scores in this population.

Two critical aspects of this study are worth highlighting: it was conducted *in vivo* and there were no negative effects of the medication on vaginal lactobacilli. However, there are limitations to consider. The study did not include a control group, as any neutral product applied to the vaginal environment could potentially alter the local microbiota. Instead, we used a before-and-after design, where each participant served as her own control. In a previous study by Srinivasan et al [34], the authors compared changes in the vaginal microbiota, metabolome, and pH among women using low-dose vaginal estradiol tablet or low pH moisturizer gel for 12-weeks vs placebo. They observed that there was a decrease in pH from baseline to 12-weeks within placebo group. The study by Mitchell et al [35] also showed changes in the vaginal environment in relation to the control group.

Additional limitations include the timing of specimen collection, which was not standardized to specific menstrual cycle phases since participants attended the clinic based on availability; the fact that the specimen collection of vaginal material for microbiota analysis was collected from the vaginal cul-de-sac with a sterile swab, as this was the orientation of the laboratory that performed the analyzes; and the fact that the gel used also includes glycogen in its composition. Glycogen is a sugar stored in vaginal wall cells, serving as a crucial carbon and energy source for vaginal bacteria. Its production is influenced by estrogen. When released into the vaginal environment, glycogen is broken down into glucose, maltose, and malto-oligosaccharides by human and/or bacterial amylases. Lactobacilli are among the species that consume glycogen, converting it into lactic acid, thus helping to maintain the vaginal environment acidic. Therefore, glycogen indirectly contributes to protecting vaginal health [36].

Our findings underscore that even asymptomatic women can have vaginal dysbiosis, which may increase the risk of gynecological complications [10-13], and, in women with human papillomavirus (HPV), a higher risk of cervical carcinogenesis [12]. Therefore, maintaining a physiological vaginal pH, inhibiting the growth of pathogenic bacteria, and maintaining a balanced vaginal environment is essential for women’s health [2,3]. Based on our results, we suggest that lactic acid-containing gels could be used in women with episodic and recurrent bacterial vaginosis, as maintenance therapy, aiming to reduce recurrences [37,38]. Additionally, women with type IV CSTs may benefit from this product when trying to prevent possible complications of vaginal dysbiosis. However, additional studies need to be performed to support the use of lactic acid-containing gels for these indications.

## Conclusions

With the caveat that this study did not include a placebo control, our data suggests that the lactic acid-containing gels showed a significant reduction in vaginal pH values and Nugent scores in asymptomatic women, indicating possible improvement in vaginal health. Although the intervention did not influence microbial diversity and the type of vaginal microbiota at the end of the study, it successfully reduced the prevalence of specific taxonomic groups considered unfavorable for vaginal health, highlighting its potential role in promoting and maintaining a healthy vaginal environment.

## Supporting information

S1 Table Classification data on microbiota type, pH value and Nugent score for each participant (N=55). (DOCX)

S2 Lactic acid gel acceptability questionnaire. (PDF)
