# Supplementary material for: In vivo assessment of the effect of gel containing lactic acid and glycogen on vaginal microbiota and pH of asymptomatic women of reproductive age
Source: PLoS One. 2025 Apr 24;20(4):e0321737. doi: 10.1371/journal.pone.0321737 (PMC12021183; doi:10.1371/journal.pone.0321737)
Supplement: S1 Table — (DOCX) [file pone.0321737.s001.docx]

**Supplementary Table 1: Classification data on microbiome type, pH value and Nugent score for each participant (N=55).**

| **Patient code** | **Microbiota type** | | **pH** | | **Nugent** | |
| --- | --- | --- | --- | --- | --- | --- |
|  | **T=0** | **T=1** | **T=0** | **T=1** | **T=0** | **T=1** |
| P01 | IV | IV | 5 | 5 | 8 | 0 |
| P02 | I | I | 5 | 6 | 8 | 0 |
| P03 | I | I | 4 | 4 | 0 | 0 |
| P04 | IV | III | 4 | 4 | 0 | 0 |
| P05 | III | I | 4 | 5 | 0 | 0 |
| P06 | III | IV | 5 | 5 | 8 | 8 |
| P07 | I | I | 4 | 4 | 0 | 0 |
| P08 | I | I | 5 | 4 | 0 | 0 |
| P09 | IV | IV | 4 | 4 | 8 | 8 |
| P13 | III | III | 6 | 5 | 8 | 0 |
| P16 | IV | III | 5 | 5 | 0 | 0 |
| P17 | IV | IV | 5 | 5 | 8 | 5 |
| P18 | III | I | 4 | 4 | 0 | 0 |
| P19 | III | III | 5 | 4 | 0 | 0 |
| P20 | IV | IV | 6 | 4 | 8 | 5 |
| P21 | I | I | 4 | 5 | 0 | 0 |
| P22 | III | IV | 6 | 5 | 8 | 8 |
| P23 | I | I | 5 | 4 | 0 | 0 |
| P24 | I | I | 4 | 4 | 0 | 0 |
| P26 | II | III | 5 | 4 | 0 | 0 |
| P27 | IV | IV | 5 | 5 | 8 | 8 |
| P28 | III | I | 5 | 4 | 0 | 0 |
| P29 | III | III | 4 | 4 | 0 | 0 |
| P30 | IV | IV | 5 | 5 | 8 | 8 |
| P31 | III | III | 5 | 4 | 1 | 1 |
| P32 | III | IV | 4 | 4 | 0 | 8 |
| P33 | II | IV | 5 | 5 | 1 | 0 |
| P34 | N/A | I | 4 | 4 | 8 | 0 |
| P35 | V | V | 4 | 4 | 0 | 0 |
| P36 | I | I | 4 | 4 | 0 | 0 |
| P38 | IV | IV | 6 | 5 | 8 | 8 |
| P39 | III | III | 4 | 4 | 0 | 0 |
| P40 | IV | IV | 5 | 5 | 5 | 5 |
| P41 | IV | IV | 4 | 5 | 8 | 8 |
| P42 | III | V | 4 | 4 | 0 | 0 |
| P43 | I | I | 5 | 4 | 0 | 0 |
| P44 | IV | IV | 5 | 4 | 8 | 0 |
| P45 | III | III | 5 | 4 | 1 | 0 |
| P46 | IV | III | 5 | 5 | 1 | 1 |
| P47 | I | I | 4 | 4 | 0 | 0 |
| P48 | IV | I | 5 | 4 | 8 | 0 |
| P49 | I | I | 4 | 4 | 0 | 0 |
| P50 | IV | IV | 5 | 5 | 8 | 8 |
| P53 | IV | I | 6 | 5 | 8 | 0 |
| P54 | IV | III | 5 | 4 | 0 | 0 |
| P55 | III | III | 4 | 4 | 0 | 0 |
| P56 | III | III | 4 | 4 | 5 | 0 |
| P57 | III | III | 5 | 4 | 5 | 0 |
| P58 | IV | IV | 5 | 4 | 8 | 0 |
| P59 | I | I | 4 | 4 | 0 | 0 |
| P60 | IV | IV | 5 | 5 | 8 | 8 |
| P61 | I | I | 5 | 5 | 5 | 0 |
| P62 | IV | III | 4 | 4 | 0 | 0 |
| P63 | III | I | 4 | 4 | 5 | 0 |
| P64 | I | III | 4 | 4 | 0 | 0 |
