# Supplementary material for: In vivo assessment of the effect of gel containing lactic acid and glycogen on vaginal microbiota and pH of asymptomatic women of reproductive age
Source: PLoS One. 2025 Apr 24;20(4):e0321737. doi: 10.1371/journal.pone.0321737 (PMC12021183; doi:10.1371/journal.pone.0321737)
Supplement: S2 — (PDF) [file pone.0321737.s002.pdf]

Appendix 2: Lactic acid gel acceptability questionnaire

Name: ______________________________________ **Date of birth:** _____/_____/ _____

1. Regarding the **LUBRICATION / MOISTURE** of your intimate area, would you say that:

( ) It has improved a lot

( ) Improved

( ) No change

( ) It got worse

( ) It got a lot worse

Why?

____

____

2. Regarding the **DRYNESS** of your intimate region, would you say that:

( ) It has improved a lot

( ) Improved

( ) No change

( ) It got worse

( ) It got a lot worse

Why?

____

____

3. During the study period, did you have sexual relations?

( ) Yes. On average, how many times a week? ____________________________

( ) No.

4. Regarding **COMFORT** during your sexual relations, would you say that:

   ( ) It has improved a lot

   ( ) Improved

   ( ) No change

   ( ) It got worse

   ( ) It got a lot worse

   Why?

   ____

   ____

5. This product is available on the market: how likely are you to buy and use the product? Considering the following scoring scale, 0 you would not use it and 10 you would definitely use it:

   ( )0 ( )1 ( )2 ( )3 ( )4 ( )5 ( )6 ( )7 ( )8 ( )9 ( )10

6. This space is free, leave your comments, criticisms or suggestions:

____

____

____

____

____

____________ Participant's rubric ____________ Researcher rubric
